# A linear programming computational framework integrates phosphor-proteomics and prior knowledge to predict drug efficacy

**DOI:** 10.1186/s12918-017-0501-6

**Published:** 2017-12-21

**Authors:** Zhiwei Ji, Bing Wang, Ke Yan, Ligang Dong, Guanmin Meng, Lei Shi

**Affiliations:** 1 School of Electronical and Information Engineering, Anhui University of Technology, Maanshan, 243002 China; 2 School of Information & Electronic Engineering, Zhejiang Gongshang University, 18 Xuezheng Road, Hangzhou, 310018 China; 3 College of Information Engineering, China Jiliang University, 258 Xueyuan Streat, Hangzhou, 310018 China; 4 Department of Clinical Laboratory, Tongde Hospital of Zhejiang Province, 234 Gucui Road, Hangzhou, 310012 China

**Keywords:** Treatment effect, Compound, Signaling pathway, Linear programming, Prior knowledge

## Abstract

**Background:**

In recent years, the integration of ‘omics’ technologies, high performance computation, and mathematical modeling of biological processes marks that the systems biology has started to fundamentally impact the way of approaching drug discovery. The LINCS public data warehouse provides detailed information about cell responses with various genetic and environmental stressors. It can be greatly helpful in developing new drugs and therapeutics, as well as improving the situations of lacking effective drugs, drug resistance and relapse in cancer therapies, etc.

**Results:**

In this study, we developed a Ternary status based Integer Linear Programming (TILP) method to infer cell-specific signaling pathway network and predict compounds’ treatment efficacy. The novelty of our study is that phosphor-proteomic data and prior knowledge are combined for modeling and optimizing the signaling network. To test the power of our approach, a generic pathway network was constructed for a human breast cancer cell line MCF7; and the TILP model was used to infer MCF7-specific pathways with a set of phosphor-proteomic data collected from ten representative small molecule chemical compounds (most of them were studied in breast cancer treatment). Cross-validation indicated that the MCF7-specific pathway network inferred by TILP were reliable predicting a compound’s efficacy. Finally, we applied TILP to re-optimize the inferred cell-specific pathways and predict the outcomes of five small compounds (carmustine, doxorubicin, GW-8510, daunorubicin, and verapamil), which were rarely used in clinic for breast cancer. In the simulation, the proposed approach facilitates us to identify a compound’s treatment efficacy *qualitatively* and *quantitatively*, and the cross validation analysis indicated good accuracy in predicting effects of five compounds.

**Conclusions:**

In summary, the TILP model is useful for discovering new drugs for clinic use, and also elucidating the potential mechanisms of a compound to targets.

**Electronic supplementary material:**

The online version of this article (10.1186/s12918-017-0501-6) contains supplementary material, which is available to authorized users.

## Background

In recent years, molecular and cellular biology has significantly improved our understanding of cancers in human, however, the discovery and use of therapeutic drugs still faces major challenges [1]. Particularly, insufficient understanding of pathological and therapeutic mechanisms at a cellular level holds back the growth of the number of new drugs taken for clinical use [2]. More difficulties arise to predict the exact effects of those approved drugs [3]. As a result, patients with serious illnesses are left with few treatment options; and the new medicines are usually extremely expensive. Only 10% of drug molecules entering clinical trials succeed and approximately 3 out of 10 drugs generate enough profit to pay back the investment [4]. The linkage between basic science and useful treatment has to be built based on deep understanding of biological networks, as well as drug-induced molecular changes in these networks [5]. A global view covering the interconnectivity of the signaling proteins and their functional contributions to cancer development is critical for the success of targeted drug anti-cancer therapies. Accordingly, the systems biology approaches, which combine experimental data and mathematical modeling, provide an effective framework to understand cancers and develop new drugs [6].

Recently, the public LINCS genomic database (Http://www.lincsproject.org/) was established, aiming to create a network-based understanding of biology by cataloging molecular changes in gene expression and signal transduction occurring when cells were exposed to various genetic or drug perturbations [7]. The LINCS is helpful for drug screen and discovery via better understanding intracellular pathways. Particularly, the P100 [8] data in LINCS project, is mass spectrometry (MS) based phosphor-proteomic profiles caused by a wide range of small molecule chemical compounds on several representative cancer cell lines, such as MCF7 (breast cancer) and PC3 (prostate cancer) cell lines [9]. Due to unclear targets or treatment effects, most part of compounds used in P100 is not widely applied as drugs in market. Identifying the treatment effects of a compound on certain cell line is of great significance of discovering new potential drugs and improving the response to therapies in clinic.

Considering the P100 data [10] was only measured at 6 h after compound perturbations, it is impossible to build ODE-based model of intracellular pathway network to predict drug efficacy [6]. Mitsos and coworkers firstly proposed an integer linear programming method (ILP) to predict drug treatment effect with phosphor-proteomic data, however, their model can only address the signaling networks with simple topology [11]. We recently developed a Binary Linear Programming based approach (BLP) for modeling large-scale signaling pathways [10]. BLP was applied on LINCS dataset (L1000 gene expression profiles [12] and P100 proteomics [10]) to identify compound treatment effects via pathway alterations. Different from the ILP approach [11], our BLP model can address the signaling networks which have complex topologies. However, BLP shows obvious disadvantage: 1) the definition of the state of each protein node with Boolean variable is not sufficient enough to represent the relative changes of the proteins between treatment and un-treatment; 2) BLP model may lead to biased predictions due to the division of the training and testing set of P100 data is fixed in advance.

**Fig. 1:**
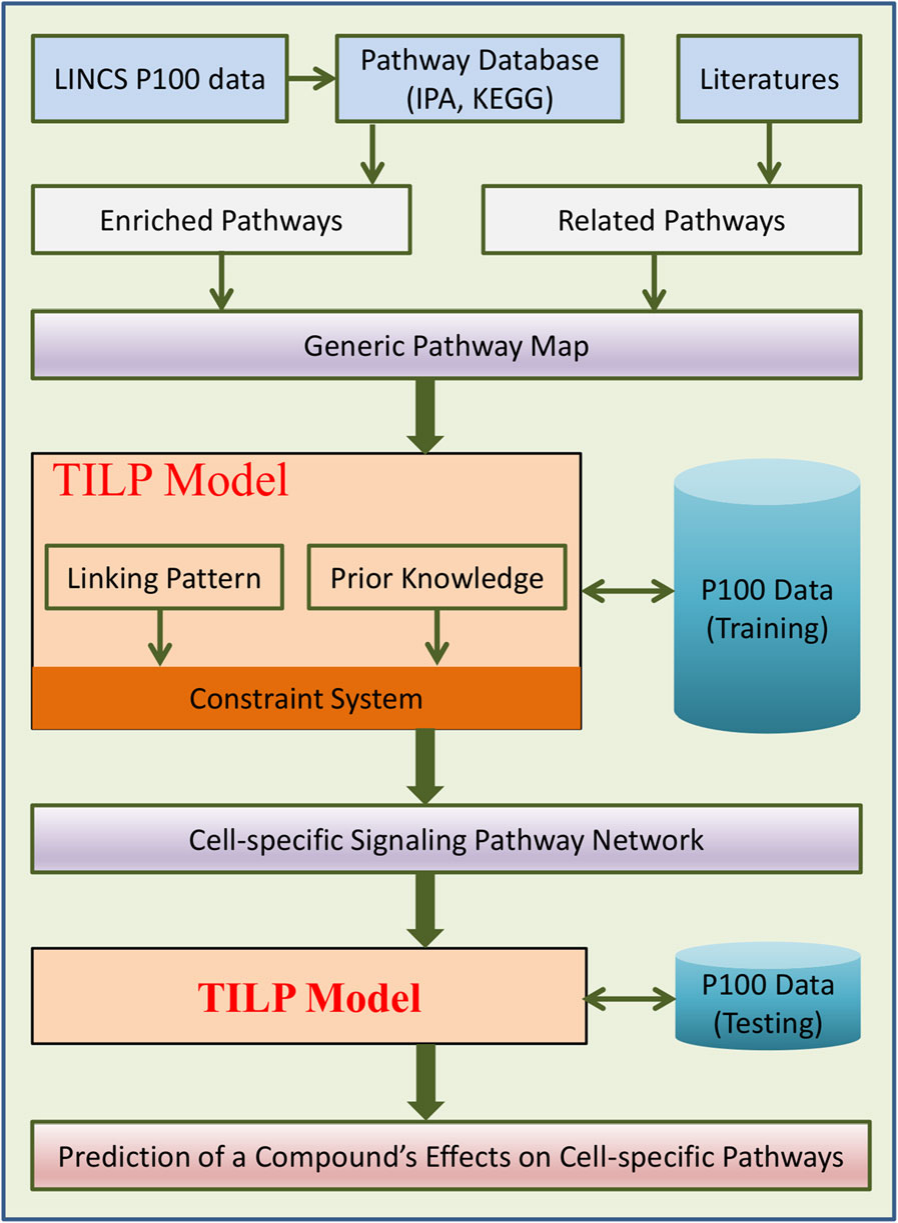
The flowchart of the TILP model

In this paper, we proposed a novel systemic modeling approach, namely Ternary status based Integer Linear Programming (TILP), to infer cell-specific signaling pathway network and predict the response of cells to new compounds (Fig. 1). The Ternary status for signaling proteins and the prior knowledge about potential targets with constraints, make the TILP model more representative to describe the dynamic changes (e.g. down-regulation, up-regulation, and no-change) of the response network under perturbed conditions. To validate the performance of the proposed model TILP, the P100 data related with MCF7 cell line was used in the simulation. First, we manually built a generic pathway map related with MCF7 cells; and then infer MCF7-specific pathway by using TILP on the training dataset of ten compounds. Then, TILP was applied to re-optimize the inferred cell-specific pathway using the dataset of five testing compounds, and *qualitatively* and *quantitatively* predict their treatment effects on MCF7 cell line. The cross-validation indicates high accuracy in predicting the effects of five testing cases. In summary, the proposed computational model was capable of elucidating potential molecular mechanisms of a chemical compound’s efficacy.

## Results

### Experimental data

The P100 phosphoproteomic data for MCF7 breast cancer cell line was considered, which was treated by 15 small molecule chemical compounds (see Table 1). These 15 compounds can be considered as 15 perturbed conditions to MCF7 cell line. The first 10 compounds have been proved to be the potential anticancer drugs to treat breast cancer; the remaining 5 compounds were rarely studied for this disease. In this study, two subsets of the P100 phosphoproteomic data were analyzed. The first subset with 10 compounds was used to infer the specific signaling pathways of MCF7 cells using the TILP method. The second subset with 5 compounds was used to predict drug treatment effects by re-optimizing the inferred cell-specific network using TILP.

**Table 1:** P100 data on 15 chemical compounds were used in this study

No.	Chemical compound	Treatment concentration (uM)
1	Fulvestrant	1
2	Paclitaxel	1
3	Staurosporine	1
4	Irinotecan	100
5	Scriptaid	10
6	Anisomycin	15
7	Trichostatin	1
8	MS-275	10
9	Digoxin	5.2
10	Geldanamycin	1
11	Carmustine	10
12	Doxorubicin	6.8
13	GW-8510	10
14	Daunorubicin	1
15	Verapamil	15

The expression of each protein (*ρ*) in P100 dataset was represented as log2 ratio of treatment to control (un-treatment). Therefore, the raw data of P100 were normalized to ternary values (1, 0, and −1) with threshold 1.2, where 1 corresponds to up-regulation (*ρ* ≥ log_2_(1.2)), 0 to no-change (−log_2_(1.2) < *ρ* < log_2_(1.2)), and −1 to down-regulation (*ρ* ≤  − log_2_(1.2)). The normalization method reflects the relative changes on signaling proteins after compound perturbation.

### Constructing a generic pathway map

**Fig. 2:**
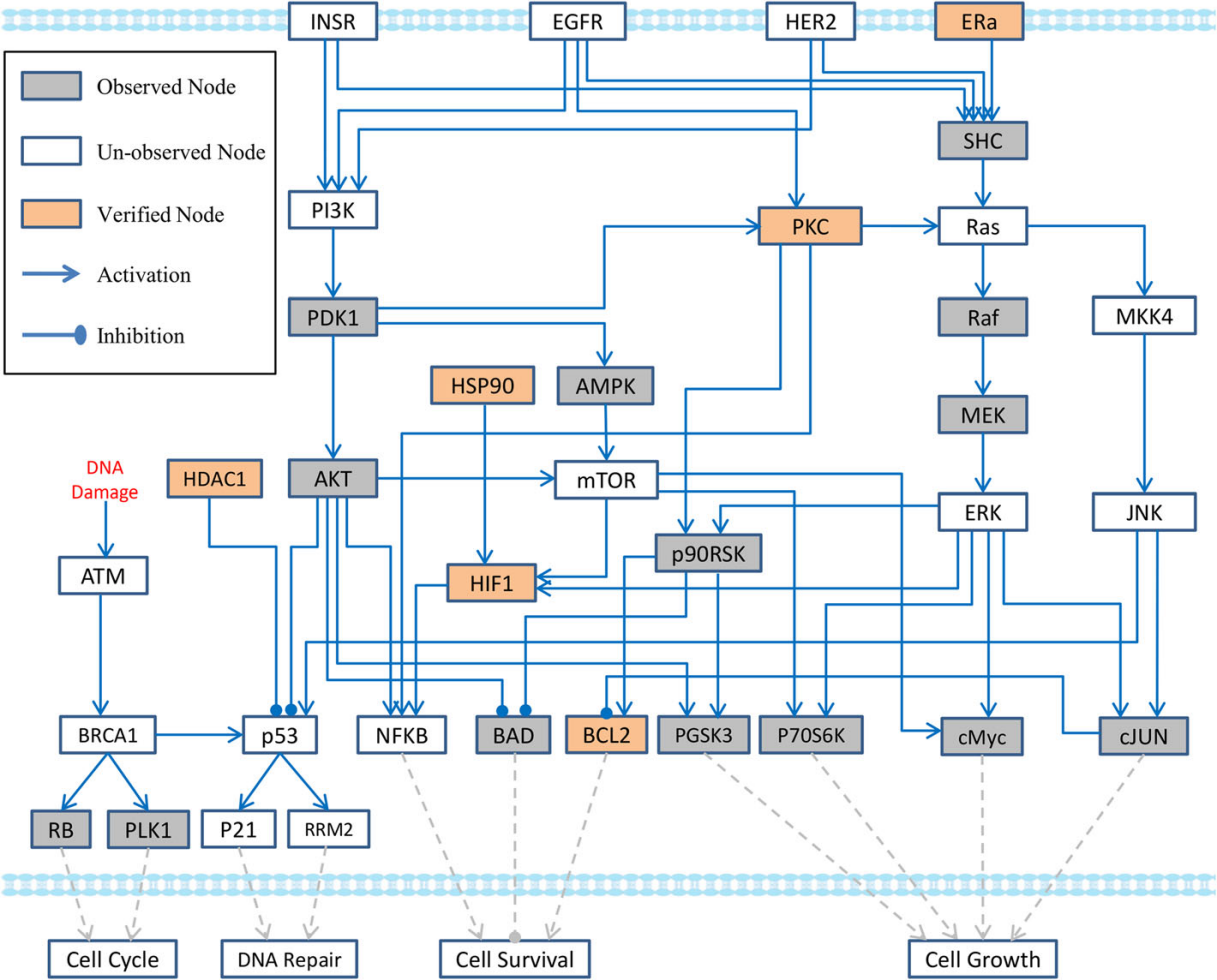
MCF7-related generic pathway map

Based on the threshold described in above section, we selected the differentially expressed proteins (DEPs) from the preprocessed P100 dataset, and imported them into a public platform of pathway analysis, IPA (http://www.ingenuity.com). Combining all the top-ranked enriched signaling pathways (*P* value < 0.01) from IPA and the MCF7-related pathways widely reported in previous literatures, we manually constructed a generic pathway map (see Fig. 2) that includes important signaling pathways in breast cancer cells, such as ER/SHC pathway [13], EGFR signaling [14], HER2 signaling [15], INSR pathway [16], and HDAC pathway [16] et al. For example, several evidences proved the interplay between BRCA1 and cell cycle regulation. Particularly, BRCA1 plays an important role in controlling DNA damage induced G2/M checkpoint [17]. HSP90 is a potential novel breast cancer cell biomarker, which participates in primary tumor growth [18]. Previous studies suggested that PKC could act as a positive regulator of growth in humor-derived mammary cells, potentially through activating Ras signaling-regulated pathway [19]. Activation of tumor suppressor p53 can induce cell cycle arrest for repairing DNA-lession [20]. NFKB has shown the capability of enhancing cell survival and anti-cancer drug resistance [21]. Particularly, the outcome of this pathway network mainly associated with four cell functions, including cell survival, and cell growth, cell cycle regulation, and DNA repair.

There are 35 nodes and 50 directed edges in the generic pathway map shown in Fig. 2. Nodes in the network represent signaling kinases with their discrete values: −1, 1, or 0, where −1, 1, and 0 stand for “down-regulation”, “up-regulation”, and “no-change”, respectively. These values represented possible states of proteins after the tumor cells were treated by compounds. We also presented two types of directed edges in the signaling network topology: inhibition  or activation  . Each edge in the network model was thus encoded by an integer parameter, which had two possible values: −1 (inhibition) or. 1 (activation). After receiving the perturbation with certain compound, the state of each signaling reaction is denoted with logical values 0 or 1, indicating “occur” or “does not occur”. Moreover, the nodes with grey color were observed in the P100 assay, and the remaining nodes were un-observed. Particularly, the nodes with orange color were proved as the targets of certain compounds in literatures (Table 2), which provide complementary knowledge to current p100 data. In addition, the states of four output nodes represent key cell functions, which were determined by their upstream parent nodes (Transcriptional Factors, TFs) locating at the end of signaling pathways. A “cell function” node is up-regulated if and only if at least an upstream positive TF is up-regulated or a negative TF is down-regulated. We used OR gates to model the effect of TFs on the changes of cell functions. In our generic pathways, four cell function nodes were calculated with following Eq. (1–4).

1 $$ {F}_{cell\  Cyc}= RB+ PLK1 $$

2 $$ {F}_{DNA\  Rep}=P21+ RRM2 $$

3 $$ {F}_{cell\  Sur}= NFKB- BAD+ BCL2 $$

4 $$ {F}_{cell\  Gro}= pGSK3+p70S6K+ cMYC+ cJUN $$

**Table 2:** The compound targets were proved in the previous literatures

NO.	Compound treatment	Up-regulation	Down-regulation	PMID
1	Fulvestrant		ER	26,272,024
2	Paclitaxel	JNK	BCL2	22,433,870
3	Staurosporine		PKC	17,171,646
4	Irinotecan	HIF1, NFKB	ER	24,966,994
5	Scriptaid		HDAC1	14,620,913
6	Anisomycin		BCL2	23,261,849
7	Trichostatin		HDAC1	24,626,188
8	MS-275		HDAC1	18,579,665
9	Digoxin		HIF1	19,020,076
10	Geldanamycin		HSP90	26,674,599, 26,405,178

According to Eq. (1–4), four cell functions (cell cycle regulation, DNA repair, cell survival, and cell growth) are quantified as integer variables, and are calculated from their upstream TFs (see Methods).

**Fig. 3:**
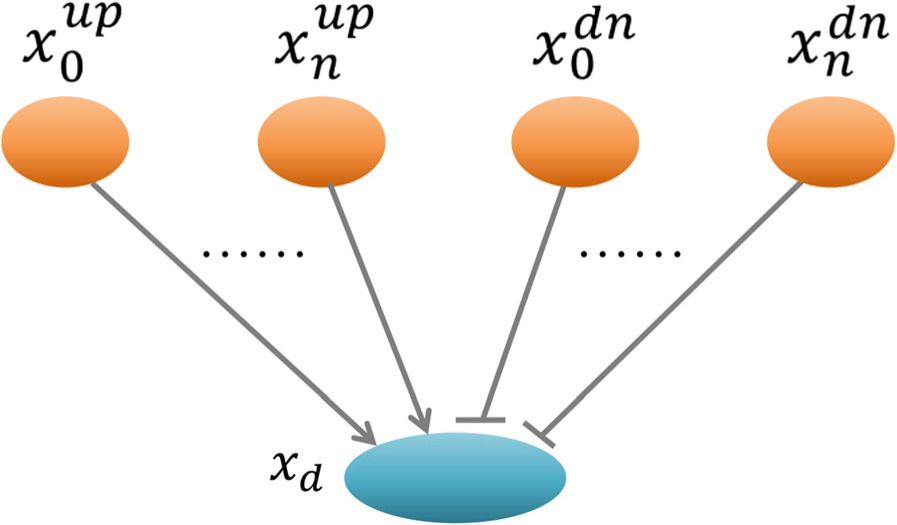
The linking pattern of protein nodes, which is common in the signaling network

Moreover, we defined a linking pattern of protein nodes, so that a complicated signaling network can be represented with this pattern (Fig. 3). In this model, the states of protein nodes and connected edges (regulations) were all constrained with our developed mathematical rules (Additional file 1: Text S1). Moreover, all the regulations, connected to the same protein, were considered as independent. In other word, a child (downstream) node can be regulated by at least one of parent (upstream) nodes simultaneously, and its status was finally determined by all of its parent nodes and the linking pattern. Our TILP model combined P100 data with prior knowledge, optimizes the generic pathway map by removing inconsistent links from the original network. The inference of the states of all the nodes and edges in the network was implemented with a set of linear constraints in TILP. The details regarding the mathematical constraints were described in Additional file 1.

### Inferencing a cell-specific pathway network by TILP

In order to infer the cell-specific network from our constructed generic pathway map that best reproduces the experimental data, we minimized the differences between the model’s predicted values and the corresponding observational values for the subset of protein nodes that are observed, as well as the complexity of network structure. To solve the multi-objective optimization problem, we developed a TILP model of the generic pathway network, and obtained a cell-specific pathway network by optimizing formula (9) shown in Methods. The idea behind TILP is that the states of nodes are normalized to ternary numbers; directed edges between two connected nodes are represented as Boolean numbers; and linear constraints are defined to describe the casual relationship between child and parent nodes. The prior knowledge shown in Table 2 was represented as linear constraints to guide the optimization process. The TILP model of a signaling network was solved by using MATLAB toolbox Gurobi 6.5.1 [22]. The toolbox guarantees to minimize a global error function that combines the requirements of data fit and simplicity. The fitting precision of the inferred specific pathways of MCF7 cells was 83.33%, which demonstrates that TILP fits well on P100 data. The definition of fitting precision is described in Additional file 1: Text S2.

**Fig. 4:**
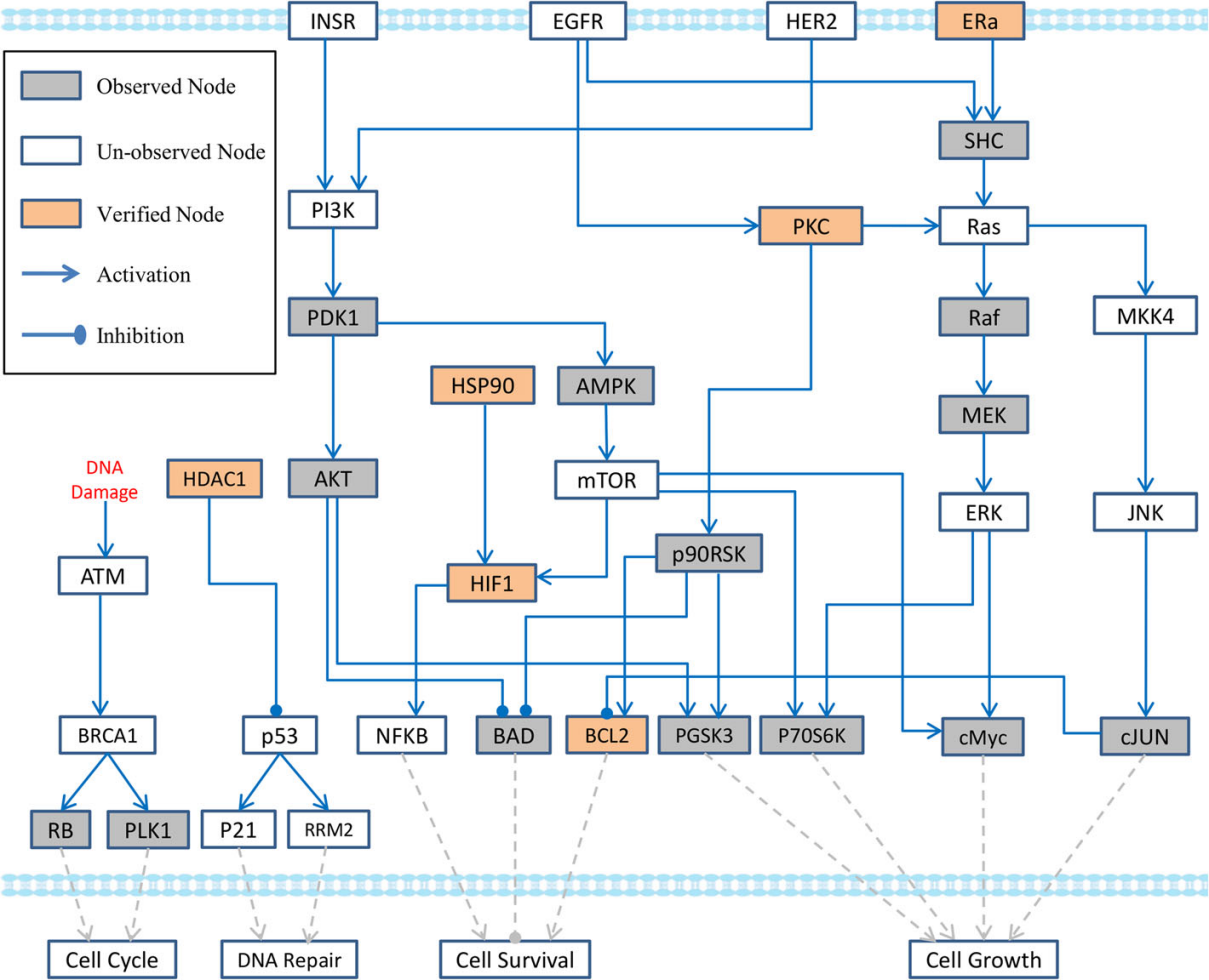
The MCF7-specific signaling pathway network inferred by TILP model

The inferred MCF7-specific pathway networks are shown in Fig. 4. After the TILP model optimizes above two objectives with P100 data, we keep those edges whose regulation occurs for some treatment conditions, and remove the edges whose regulations completely do not occur for all the conditions. Comparing with the signaling pathway network shown in Fig. 2, totally 13 redundant edges were removed from the generic pathway map due to the inconsistencies between these links and observations of connected nodes. For example, both BRCA1 and p53 are tumor suppressors and are involved in a number of cellular processes including cell cycle arrest, apoptosis, and DNA damage repair. Dong*,* et al. have identified that there may be a functional link between p53 and BRCA1 in DNA double strand breaks repair process at transcriptional and possible post-transcription level; however, the association between them is still unclear [23]. In our study, the result of optimization shows that, the link BRCA1→p53 does not exist in the inferred MCF7-specific pathway network (Fig. 4). In addition, four output nodes associated with four types of cell functions, which quantitatively represent the compound-induced treatment effects. To prove the reliability of the cell-specific pathway network inferred by TILP, cross-validation was described in detail in the section “Discussion”.

### Predicting a compound’s treatment effects

**Fig. 5:**
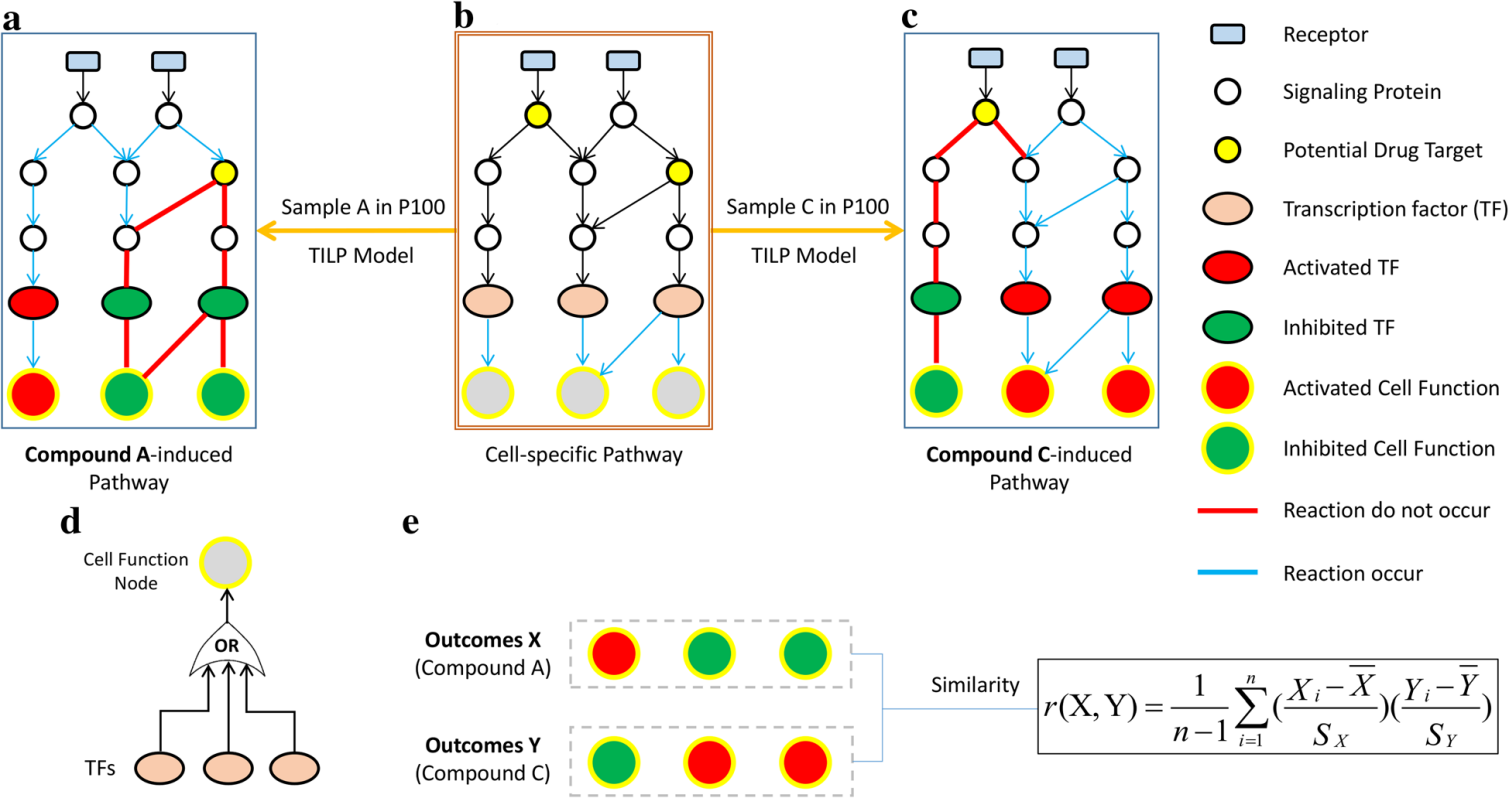
**a** compound A-induced response network; **b** Cell-specific network; **c** compound C-induced response network; **d** OR gates used to model output nodes; **e** the calculation of similarity between two compounds

Our *assumption* is that the topology of cell-specific signaling network are stable if there is no perturbation from its microenvironment on the tumor cells. Once a small compound acts on tumor cells, some intracellular signaling pathways will represent significant changes (up-regulation or down-regulation) to response this external perturbation, which subsequently alters their cell functions or behaviors. Here, we firstly used TILP approach to fit the generic pathway map with the first part of phosphorylation data (10 training compounds) in P100 and obtain a cell-specific pathway network. The second part of data (5 testing compounds) was fitted to optimize the network model of cell-specific pathways, and the states of all the phosphoproteins and signaling reactions can be inferred so that the compound’s effects is easily identified. As shown in Fig. 5, we predicted a compound’s treatment effects with two aspects: 1) qualitatively identify the treatment effects via compound-induced pathway alterations. Considering the response network treated by a compound will induce activation of some reactions, comparing it with the cell-specific pathway network facilitates us to easily identify the treatment effects according to the pathway alterations: down-regulation, up-regulation, and no-change. For example, the comparison between Fig. 5a and b facilitates us to identify the treatment effects of compound A on cancer cells. 2) quantitatively evaluate the similarity of treatment effects between two compounds according to the outcome vectors of signaling network. The extracellular perturbations on cancer cells finally reflects the changes of cell functions through signal transduction; therefore, the expression of all the output nodes in the signaling network provides an effective way to quantitatively represent the efficacy of compound treatment. In our work, Correlation Coefficient [24] was applied to calculate the similarity between two compounds with their corresponding vectors consisting of four output nodes. The similarity of treatment effects between compound A and C (Fig. 5a and c) can be calculated with the output vectors via formula (12) in Methods. The regulation of multiple TFs on a same cell function is defined as logical OR gate (Fig. 5d), which indicates that different TFs are independent.

Additional file 2 Figure S1, Additional file 3: Figure S2, Additional file 4: Figure S3, Additional file 5: Figure S4 and Additional file 6: Figure S5 present the response network perturbed by 5 testing compounds: carmustine, doxorubicin, GW-8510, daunorubicin, and verapamil (Table 1). For all 15 measured proteins in the MCF7-specific pathway network, fitting precision of these observed nodes for five compounds were 100%, 80%, 86.67%, 93.33%, and 80%, respectively. According to the pathway alterations, we can qualitatively identify the compound-induced treatment effects on MCF7 cells. From Additional file 2: Figure S1, Additional file 3: Figure S2, Additional file 4: Figure S3, Additional file 5: Figure S4 and Additional file 6: Figure S5, the reactions with red color represent up-regulation, green is for down-regulation, and black is for no-change. The reactions with yellow color did not occur after compound treatment

I. *Carmustine* [25], a potential anticancer agent, activated the MAPK (MEK/ERK, and JNK) pathway via ER→SHC→RAS, and inhibited PI3K/AKT, HSP90/HIF, and AMPK pathways in MCF7 (see Additional file 2: Figure S1). An’s work proved that carmustine induced phosphorylation of the MAPKs, such as ERK, JNK, and p38, etc. [26].
II. *Doxorubicin* [27], the current front-line therapy for breast cancer, not only activated MAPK pathway as carmustine, but also up-regulated EGFR/PKC pathway. In addition, treatment with Doxorubicin also decreased the expression of ATM/BRCA1, HSP90/NFKB, and PI3K/AKT/BAD pathways (see Additional file 3: Figure S2). Rojo and coworkers proved that ex vivo exposure of breast tumors to doxorubicin down-regulated MKP-1, and up-regulated p-ERK1/2 and p-JNK, in the majority of cases [28]. Liu, et al. also found that the active TGFβ signaling exhibited resistance to the DNA-damaging agent doxorubicin in some breast tumors [29], and TGFβ induces a DNA-repair deficiency through downregulating DNA-repair genes, such as ATM, and BRCA1 [30].
III. Additional file 4: Figure S3 indicates that *GW-8510* potentially inhibited most of signaling pathways in the MCF7-specific network expect increase the expression of HDAC1.
IV. *Daunorubicin* [31], a market selling heterocyclic anticancer drug, mainly activate MAPK (MEK/ERK, and JNK) pathways via ER→SHC→RAS, and down-regulated cell survival through reduced PI3K/AKT pathway in the inferred MCF7-specific pathway network (Additional file 5: Figure S4). Stulpinas and colleagues observed that daunorubicin treatment induced the activation of JNK and phosphorylation of its direct target c-Jun along with inactivation of AKT [32]. We also noticed from Additional file 5: Figure S4 that, the activation of HDAC1 induced the down-regulation of p53. D’Assoro’s study indicates that treatment on MCF7 cell line with daunorubicin resulted in the arrest of both G1/S cell cycle progression [33].
V. As to V*erapamil* [34], a calcium channel antagonist, is known as a potential anticancer agent. In Additional file 6: Figure S5, this compound induced up-regulation of ATM/BRCA1 to increase the cell cycle, and down-regulation of MAPK, HSP90/HIF1 to reduce the tumor growth.

In addition, the “cell function” vector consists of four output nodes, which can be potentially used to calculate the similarity between two compounds (Fig. 5e). For example, the outcome vectors of response network, treated by Paclitaxel and Staurosporine, were [0, 0,-3,-1] and [0, 0,-2,-2], indicating that both compounds may potentially induce cell death. Compared to the topological structure of cell-specific pathway network shown in Fig. 4, the similarities of compound-induced network are 91.89% (paclitaxel), and 89.19% (staurosporine), respectively. In Yu’s work, MDA-MB-435 breast cancer cells represent similar cell apoptosis rates after treatment with paclitaxel or staurosporine [35]. Cartee, et al. also found that exposed the BG-1 cells to staurosphorine or paclitaxel exhibited similar apoptotic cell death via caspase activation [36]. Another example is the calculated outcomes of GW-8510-induced response network were similar as digoxin did, that are [−2,-2,-3,-4] and [−2,-2,-1,-4], respectively. Although the predicted inhibition effects of GW-8510 on above four cell functions cannot be confirmed, Chung and coworkers introduced that GW-8510 may have high inhibitory activities against cancer cells. They further confirmed that the novel functional links between GW-8510 and cell cycle inhibition according to the microarray experiments [37]. In summary, most of the above predictions from networks are consistent with the conclusions in previous studies, which implies that TILP model is reliable for identifying compound treatment effects.

## Discussion

In this study, we presented a novel computational modeling approach TILP to reconstruct cell-specific pathway network and predict the system’s response to compound treatment by combining prior knowledge from literature and phosphoproteomic data collected from various types of perturbation experiments. In order to test the power of TILP model, we selected a set of P100 proteomic data of 15 chemical compounds effecting on MCF7 breast cancer cell line. Based on the differential expressed proteins in P100 data, we combined all the top-ranked enriched pathways (*P* value < 0.01) from IPA with some important pathways reported in previous literatures, to manually construct a generic pathway map related with MCF7 cells, which includes 50 reactions and 35 proteins (15 proteins were observed in P100 dataset). With the TILP model for pathway modeling and optimization, a MCF7-specific pathway network of 35 nodes and 37 edges was finally inferred. Based on the topological structure of optimized MCF7-specific network, we monitored 5 cases of compound-induced response networks to predict the effects of these compounds using TILP again.

An important aspect of our approach is its practicability in inferring cell-specific network. Although continuous modeling approaches (e.g. ODEs [38]) are commonly used to model dynamics of biological networks [39]; however, parameter estimation in ODE models is a NP-hard problem [40]. TILP, a parameter-free model, based on the conception of discrete modeling, has simplified both the representation and optimization of signaling network with mathematical constraints. In the TILP model, we defined two types of constraints: (1) the states of connected proteins and reactions were defined with linear constraints; (2) the prior knowledge about the expressions of potential targets corresponding to perturbed compounds was also represented as linear constraints. Thus, the developed constraint system simplifies the optimization process and quickly searches an optimal solution from an allowable subspace. For inferencing the MCF7-specific pathway network, the optimization procedure of TILP model produced about 11,357 constraints and 4950 variables (4600 binary variables, and 350 integer variables). Totally, 4317 constraints and 2032 variables were automatically generated for predicting a compound’s effects. The TILP model is scalable to more complex signaling networks; the efficiency can still be guaranteed because a linear programming problem usually can be solved in linear time when the dimensionality is fixed [41].

Another important aspect of our approach is to help identify compound treatment effects in two ways: (1) identify the treatment effects through network topological alterations; (2) calculate the similarity of two compounds with their corresponding outcome vectors from pathway network. As described in the experimental results, most of predictions were consistent with the previous studies in the literature. However, there are still a small part of predictions could not be proved from literature, such as, GW-8510, which means it has never been used for breast cancer treatment. In addition, some evidences on other cell lines provided supports to our findings. For example, carmustine enhances chemotherapeutic efficacy by attenuating AKT activity in gliomas [42]. As a protective agent, verapamil [43] was found to significantly reduce the expression of HIF-1 alpha in many cells [44]. Liu and coworkers work also provided evidence for a crucial role for the over expression of MEK/ERK pathway in protecting kidney SP cells from ischemic/hypoxic injury, and Verapamil treatment reversed MEK-induced cell viability [45]. Further biological experiments (e.g. Western Blot, or Mass Spectrometry) will potentially validate the effectiveness of your model.

Comparing to our BLP model, the proposed TILP model successfully solved the limitations in BLP: 1) we used three states (“up-regulation”, “down-regulation”, and “no-change”) to represent the relative changes of a protein when the cancer cells received extracellular stimuli; 2) the training set is related with 10 compounds, which have been validated effectively for breast cancer treatment. And the five compounds included in the testing set are rare studied in the breast cancer therapy. Therefore, the cell-specific pathways optimized by the training set, has potential to find new drugs for cancer therapy. 3) The TILP model not only *qualitatively* identify the compound treatment effects via pathway alterations, but also *quantitatively* evaluate the similarity of a pair of compounds on the same cancer cell line.

### Cross-validation

In order to prove the reliability of our approach TILP, we tested the effects of leave-one-out cross-validation on the p100 data of the first 10 compounds which were used in model training. For each compound, we extracted phospho-proteomic data of 9 compounds to infer a cell-specific pathway network and then identify the treatment effects of the remaining one by predicting the states of all the proteins via TILP. The mean value of fitting precision for above 10 cases was 84%, suggesting that the inferred cell-specific pathway network is reliable for predicting a compound’s effects. Based on the results of the leave-one-out cross-validation we did on MCF7 cell line, we calculated the similarity of network diagrams among 10 cases (each corresponds to one compound) where each case represents a cell-specific pathway network trained from the remaining 9 compounds after leaving one compound out. In overall, the connected edges in the final inferred cell-specific pathway network (Fig. 4) are 37. The similarity of an inferred cell-specific pathway network based on the different combinations is defined as the ratio of the number of connected edges in cell-specific network of each case to the total number of connected edges (37). The mean value of the similarities of 10 cell-specific networks generated by cross validation was 99.7%. It verifies that our inferred cell-specific pathway network with p100 data of 10 compounds was reliable.

## Methods

### Experimental data

The P100 assay is a mass spectrometry-based targeted proteomic assay that detects and quantifies a representative set of ~100 phosphopeptide probes that are presented in a wide range of cell types and have been demonstrated to be modulated via perturbations https://personal.broadinstitute.org/jjaffe/p100.php. The P100 phospho-profiles are suitable for molecular signature generation, querying of signatures across cell perturbations and types, and modeling of response networks in cellular systems.

P100 data is processed through a combination of scientist review of primary data, automated quality control and statistical calculations to result in assay profiles. Currently, the signature of the assay is the complete profile of all probes measured in the assay. Five types of cells were collected and profiled, such as MCF7 (breast cancer cell), H9 (Human embryonic stem cell), et al.

In our work, we selected the validation dataset of MCF7 cell line in P100, which contains measurements of phosphor-signal treated by 26 different small compounds. The treatments were each monitored for 6 h with two replicates. All raw expression values in P100 experiment represent log2 ratios of the treatment VS. Control.

### Ternary status based integer linear programming (TILP)

In this study, the TILP computational framework was applied to systemically model a signaling pathway network with phospho-proteomic data. The developed model was used in two ways: firstly, TILP infers the cell-specific pathway network from the discrete representation of a generic pathway map; secondly, TILP predicts the treatment effects of a small compound based on the inferred cell-specific signaling network. The detail steps of the TILP framework is described as follows:

#### Discrete representation of the generic pathway map

At first, we selected the differentially expressed proteins (DEPs) from the observational data (LINCS P100). Second, we chose the top-ranked enriched signaling pathways from the database IPA (Ingenuity Pathway Analysis) based on the DEPs. Third, the generic pathway map was manually constructed by merging all the enriched signaling pathways with some important pathways studied in literature.

After determining the topological structure of the generic pathway map, the next step is the discrete representation of the signaling network. In our study, a signaling pathway map was defined as a discrete network [46], consisting of a set of nodes and directed edges. In order to reasonably describe the relative changes of cells after perturbation by a chemical compound, nodes in the network represented phosphoproteins with their discrete values 1, −1, and 0, which stand for “up-regulation”, “down-regulation”, and “no-change”(no significant change), respectively. The directed edges indicate phosphorylation reactions in the process of signal transduction induced by a compound. Here, we consider two types of reactions: inhibition  and activation  which were encoded by integer variables (−1 or +1). In our study, we defined a linking pattern of signaling proteins which was commonly seen in most of pathway network structures (Fig. 3). According to Fig. 3, we found that a child protein node can connect with several parent protein nodes, and be activated by at least one of its parents. In our TILP model, the states of all the elements (nodes and edges) in a network were satisfied to our developed mathematical constraints (see the details in Additional file 1: Text S1).

#### Inference of the cell-specific pathway network

Recently, we have developed a linear programming approach DILP to model signaling pathways with time series proteomics. In the DILP model, we proposed three states for defining the protein nodes under different time points [22]. In this study, some basic conceptions were inherited from our DILP model.

A signaling network is defined as a set of phosphoproteins *P* = {1, 2, …, *j*, …*n*_*s*_} and signal reactions *E* = {1, 2, …, *i*, …*n*_*r*_}. All the observed proteins were measured under several perturbed conditions (compound treatment), indexed by the set *C* = {*c*_1_, *c*_2_, …, *c*_*L*_}. An integer variable *x*_*j*, *k*_ *ϵ*{−1, 0, 1} indicates if the protein *j* (*j* ∈ *P*) is down-regulated (*x*_*j*, *k*_ =  − 1), up-regulated (*x*_*j*, *k*_ = 1), or un-changed (*x*_*j*, *k*_ = 0) after the tumor cells were treated by compound *k*, where *k* ∈ *C*. An output node in the network denotes a type of cell function, which is calculated from its upstream TFs. We used OR date (Fig. 5d) to model the effects of TFs on output node as a function of their expressions of connected upstream TFs:

5 $$ {F}_h={\sum}_{p=1}^{N1}{TF}_p^{+}-{\sum}_{q=1}^{N2}{TF}_q^{-} $$

where *F*_*h*_ represents the *h*-th cell function in the signaling network, ${TF}_p^{+}$ and ${TF}_q^{-}$ are the TFs that increase or reduce this cell function. A reaction *i* (*i* ∈ *E*) is represented as  (activation) or  (inhibition), where *u* and *d* are the parent (start) and child (end) nodes, respectively (*u*, *d* ∈ *P*). Moreover, the *impact* (“positive regulatory” or “negative regulatory”) of the parent node *u* on child node *d* is described as the regulation from *u* to *d* when the expression of *u* is increased or decreased. Considering that different compounds may act on different signaling pathways, a reaction may take place under some treatment conditions but not others [11]. Thus, we defined binary variable *z*_*i*, *k*_, which is 0 if reaction *i* (*i* ∈ *E*) takes place after compound *k* treatment, and 1 otherwise. The goal of this step is to detect and remove the inconsistent reactions from the topological structure of generic pathway map which do not occur in any conditions. Therefore, the binary variable *y*_*i*_ is equal to 1 if reaction *i* is removed from the generic pathway map, and 0 elsewise. Eq. (6–7) are defined to constrain the presence of reaction *i* in the optimized cell-specific network through the state of reaction *i* in the condition *k*.

6 $$ {z}_{i,k}\ge {y}_i,k\in C,i\in E $$

7 $$ 1-{y}_i\le {\sum}_{k\in T}\left(1-{z}_{i,k}\right) $$

To infer the cell-specific pathway network, we created a Ternary status based Integer Linear Programming (TILP) approach to minimize the differences between the predicted values and observational values for the subset of protein nodes that are observed, as well as the complexity of network structure. The global error function is defined as below:

8 $$ \underset{X,Y,Z}{\min}\left\{\sum \limits_{k\in T}\sum \limits_{j\in P}{\left({m}_{j,k}-{x}_{j,k}\right)}^2+\gamma \sum \limits_{i\in E}{y}_i\right\} $$

where three variable set *X*, *Y*, and *Z* denote all variables *x*_*j*, *k*_ ∈ *X*, *y*_*i*_ ∈ *Y*, and *z*_*i*, *k*_ ∈ *Z*, respectively. Eq. (8) indicates that more proteins are observed in experiment raise the accuracy of optimization. Considering the fact that experimental data often cannot cover all of the proteins in signaling network, prior knowledge extracted from literatures was represented as linear inequality to constrain the expected value of *x*_*j*, *k*_ and narrow the search space. For example, we use *x*_*j*, *k*_ ≤  − 1 to express the fact that a target has been confirmed in the previous literature: the expression of protein *j* is inhibited after tumor cells were treated by compound *k*.

In the objective function shown in Eq. (8), the first term denotes the fitting error between observed and predicted values; and the second term indicates the number of interactions in the optimized signaling network. The observed and predicted values of *j*–th protein in condition *k* were defined as *m*_*j*, *k*_, *x*_*j*, *k*_ ∈ {1, 0, −1}, respectively. When *m*_*j*, *k*_ and *x*_*j*, *k*_ are equal, the square error (*m*_*j*, *k*_ − *x*_*j*, *k*_)^2^ is equal to 0; otherwise it is either 4 or 1. Therefore, optimization of the above objective function might induce local optimal solution because of the non-uniform distribution of the term (*m*_*j*, *k*_ − *x*_*j*, *k*_)^2^. To address the bias mentioned above, a binary variable *a*_*j*, *k*_ (0 or 1) was designed as the difference between *m*_*j*, *k*_ and *x*_*j*, *k*_ as following: *a*_*j*, *k*_ will be 1, if *m*_*j*, *k*_ is not equal to *x*_*j*, *k*_, and 0 elsewise. The square error (*m*_*j*, *k*_ − *x*_*j*, *k*_)^2^ in function (8) was thus replaced by *a*_*j*, *k*_. The value of *a*_*j*, *k*_ was restricted by constraints (24–25) as shown in Additional file 1: Text S1. Finally, the above objective function (8) was simplified as formula (9).

9 $$ \underset{X,Y,Z}{\min}\left\{\sum \limits_{k\in T}\sum \limits_{j\in P}{a}_{j,k}+\gamma \sum \limits_{i\in E}{y}_i\right\} $$

where three variable set *X*, *Y*, and *Z* denote all variables *x*_*j*, *k*_ ∈ *X*, *y*_*i*_ ∈ *Y*, and *z*_*i*, *k*_ ∈ *Z*, respectively. By optimizing the above objective function, the predicted values for variable sets *X*, *Y*, and *Z* can be obtained. The negative constant *γ* in Eq. (9) is used to obtain a minimum sub-graph from the constructed generic pathway map as the finalized cell-specific signaling network ($-\frac{1}{\mid E\mid }<\gamma <0$), in which |*E*| denotes the number of interactions in the topology. In the optimization procedure, the predicted values of nodes and edges in signaling network meet the developed constraints (Additional file 1: Text S1).

**Fig. 6:**
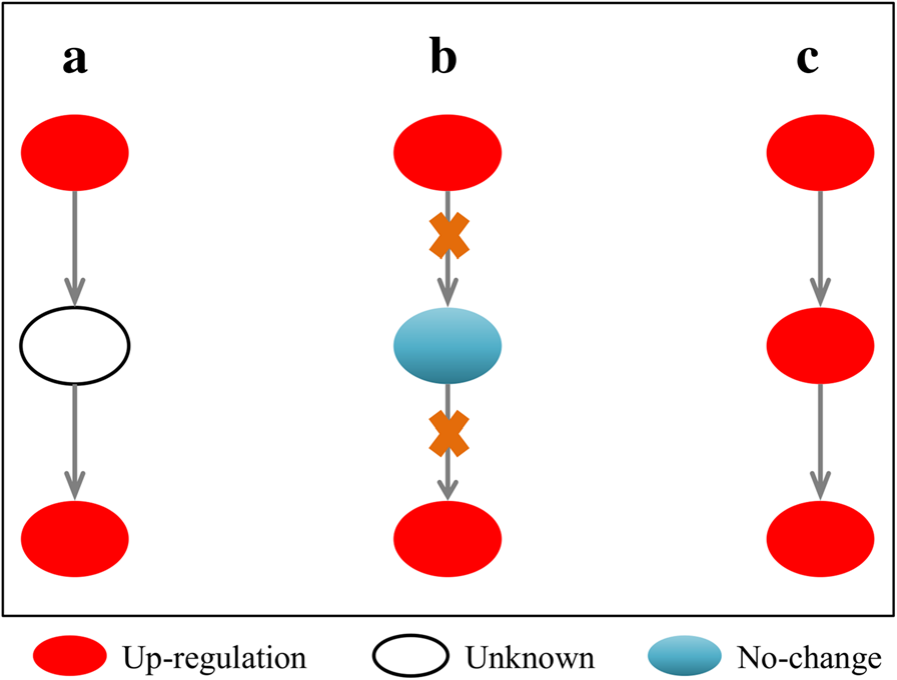
An example of adding “missing edges”. **a** a generic pathway map; **b** The cell-specific pathway network inferred by TILP when minimized the topological structure of network. **c** The cell-specific pathway network modified by “adding missing edges” with un-changed fitting precision

In Eq. (9), minimizing the complexity of network structure by edge removals might eliminate some reactions, leading to the result that some phosphor-signals may not be transduced from upstream into downstream (see one example shown in Fig. 6). Given a generic pathway map as shown in Fig.6a, we found that Fig.6b and c result in equal fitting precision between predicted and observed values. However, “missing edges” in Fig. 6b leads to interruption of the signal from upstream. Therefore, the solution shown in Fig. 6c is optimal in this case. To address the problem mentioned above, we further defined constraints to avoid missing edges in the optimization process:

10 $$ -1\le {x}_{u,k}\bullet {x}_{d,k}+{z}_{i,k}\le 1 $$

11 $$ -1\le {x}_{u,k}\bullet {x}_{d,k}-{z}_{i,k}\le 1 $$

Eq. (10) indicates that the activation reaction *i* under condition *k* takes place if *x*_*u*, *k*_ ∙ *x*_*d*, *k*_ = 1. Similarity, the inhibitory reaction *i* under condition *k* take place if *x*_*u*, *k*_ ∙ *x*_*d*, *k*_ =  − 1(Eq. (11)). Comparing with previous DILP model, TILP greatly simplifies the calculation for adding the “missing edges” in the optimization. The details were described in the Additional file 1: Text S1.

The formulations in TILP model described above were implemented in GUROBI 6.5.1 [47], which is a well-known a Matlab-based mathematical programming solver. The optimization of Eq. (9) delivers an optimal sub-network of the constructed generic pathways and best reproduces the experimental data.

#### Prediction of compound treatment effects

To predict a compound’s treatment effects, TILP model was used on the optimaml cell-specific pathway network to fit the testing data. We can identify the treatment effects of a compound from two aspects:

1. *qualitatively* identify the treatment effects via compound-induced pathway alterations. The states of all the phosphor-proteins and signaling reactions in the network could be inferred, so that we can easily find which pathway is activated (nodes are up-regulated and edges take place in this pathway), inhibited (nodes are down-regulated and edges take place in this pathway), or un-changed (the expression of nodes have no significant changes). Additional file 2: Figure S1, Additional file 3: Figure S2, Additional file 4: Figure S3, Additional file 5: Figure S4 and Additional file 6: Figure S5 provides more details about the five testing cases.
2. *quantitatively* evaluate the similarity of treatment effects between two compounds according to the outcome vectors of signaling network. The similarity score (SS) is defined as correlation coefficient, to represent the similarity of treatment effects between compound A and C with their corresponding outcome vectors of response networks.

12 $$ \mathrm{SS}=\mathrm{corrcoef}\left({T}_A,{T}_C\right) $$

where *T*_*A*_ and *T*_*C*_ are the output vectors from compound A and compound C induced response networks, respectively. Based on Eq. (12), we can easily evaluate the treatment effects between any two compounds (see the details in Fig. 5e).

## Conclusions

We developed a Ternary status based Integer Linear Programming (TILP) model to identify cell-specfic signaling pathways and predict compoun treatment effects by combining phosphorproteomics data and prior knowledge. To validate the effectiveness, we tested our TILP model on MCF7 cell line with LINCS P100 proteomics. The simulation results indicate that our model is capable of inferring the cell-specific pathway network, and further qualitatively and quantitatively predicting the compound treatment effects. For large-scale biological networks, it is difficult to estimate a large number of parameters from limited observations using ODE-based modeling approaches. As a parameter-free model, the TILP model is more efficient than continuous models (such as ODEs) because all of the elements in the network were defined as discrete variables so that the optimal solusion can be qucikly searched from an allowable subspace with constraints. Although MCF7 is used as a case of disease, the proposed model will be applicable to other disease studies with proteomic data.

## Additional files

Additional file 1: Text S1. Computational procedure: Ternary status based Integer Linear Programming (TILP). Text S2 Computational procedure: fitting precision of data (goodness of fit). (PDF 666 kb)

Additional file 2: Figure S1. The response network induced by carmustine. (PDF 472 kb)

Additional file 3: Figure S2. The response network induced by doxorubicin. (PDF 474 kb)

Additional file 4: Figure S3. The response network induced by GW-8510. (PDF 481 kb)

Additional file 5: Figure S4. The response network induced by daunorubicin. (PDF 478 kb)

Additional file 6: Figure S5. The response network induced by verapamil. (PDF 476 kb)
