# Supplementary material for: A linear programming computational framework integrates phosphor-proteomics and prior knowledge to predict drug efficacy
Source: BMC Syst Biol. 2017 Dec 21;11(Suppl 7):127. doi: 10.1186/s12918-017-0501-6 (PMC5763468; doi:10.1186/s12918-017-0501-6)
Supplement: Supplementary file 1 — Computational procedure: Ternary status based Integer Linear Programming (TILP). Text S2 Computational procedure: fitting precision of data (goodness of fit). (PDF 666 kb) [file 12918_2017_501_MOESM1_ESM.pdf]

Title page of additional files

A linear programming computational framework integrates phosphor-proteomics and prior knowledge to predict drug efficacy

Zhiwei Ji[1,2,#], Bing Wang[1,#], Ke Yan[3], Ligang Dong[2], Guanmin Meng[4], Lei Shi[2]

[1]School of Electronical and Information Engineering, Anhui University of Technology, Maanshan 243002, China

[2]School of Information & Electronic Engineering, Zhejiang Gongshang University, 18 Xuezheng Road, Hangzhou 310018, China

[3]College of Information Engineering, China Jiliang University, 258 Xueyuan Streat, Hangzhou 310018, China

[4]Department of Clinical Laboratory, Tongde Hospital of Zhejiang Province, 234 Gucui Road, Hangzhou 310012, China

[#]Corresponding authors: Zhiwei Ji (jzw18@hotmail.com), Bing Wang (wangbing@ustc.edu)

Additional files list:

Additional File 1: Text S1 Computational procedure: Ternary status based Integer Linear Programming (TILP).

Additional File 1: Text S2 Computational procedure: fitting precision of data (goodness of fit).

Additional File 2: Fig. S1 The response network induced by carmustine.

Additional File 3: Fig. S2 The response network induced by doxorubicin.

Additional File 4: Fig. S3 The response network induced by GW-8510.

Additional File 5: Fig. S4 The response network induced by daunorubicin.

Additional File 6: Fig. S5 The response network induced by verapamil.

S1: Computational procedure: Ternary status based Integer Linear Programming (TILP)

Linear Programming is a novel mathematical tool for systemic modeling and network optimization. Some previous works mainly studied the states of signaling proteins and phosphorylation events with binary variables [1], however, Boolean states ("activated" or "in-activated") are not sufficient enough to represent the variations of phosphor-signals under different perturbed conditions. In this study, we developed a Ternary status based Integer Linear Programming (TILP) approach to infer cell-specific signaling pathways using proteomic data, which were collected under various perturbed conditions with small compounds. In addition, we recently developed a linear programming approach DILP to modeling signaling pathways with time series proteomics. In the DILP model, we are the first to propose three states for defining the protein nodes under different time points [2]. Here, some basic conceptions were inherited from our DILP model.

An intracellular signaling pathway network is defined as a set of phosphoproteins $P = \{1,2,\ldots,j,\ldots n_s\}$ and signal reactions $E = \{1,2,\ldots,i,\ldots n_r\}$. All the observed proteins were measured under several perturbed conditions (compound treatment), indexed by the set $C = \{c_1, c_2, \ldots, c_L\}$. An integer variable $x_{j,k} \epsilon \{-1,0,1\}$ indicates if the protein j is up-regulated ($x_{j,k} = 1$), down-regulated ($x_{j,k} = -1$), or un-changed ($x_{j,k} = 0$) after the tumor cells were treated by compound k, in which $k \in C$. An output node in the network denotes a type of cell function, which is calculated from its upstream Transcription Factors (TFs). Here, we used OR date to model the effects of TFs on a cell function as below:

$$F_h = \sum_{p=1}^{N1} TF_p^+ - \sum_{q=1}^{N2} TF_q^- \quad (1)$$

where F_h represents the h-th cell function in the signaling network, TF_p^+ and TF_q^- are the TFs that increase or reduce this cell function. The phosphorylation reaction i ($i \in E$) can be represented as $u \rightarrow d$ (activation) or $u \dashv d$ (inhibition), where u and d are the parent (start) and child (end) nodes of this reaction, respectively ($u, d \in P$). The *impact* ("positive regulatory" or "negative regulatory") of the parent node u on child node d is described as the regulating effect from u to d when protein u is up- or down-regulated. The reaction $u \rightarrow d$ indicates that the protein u has positive regulatory role of the protein d; Similarly, the reaction $u \dashv d$ denotes that the protein u has a negative regulatory role of protein d. When the state of protein u is un-changed, u has no impact on its downstream protein d.

The sign of edge i is denoted by r_i ($r_i = 1$ for activation and $r_i = -1$ for inhibition). Considering different compounds may act on different signaling pathways, a reaction may take place under some perturbed conditions but not others. Thus, we defined binary variable $z_{i,k}$, which denotes 0 if the reaction i ($i \in E$) took place after treatment with compound k, and 1 else wise. Here, we also introduce

the binary variables $u_{i,k}^{+}$ and $u_{i,k}^{-}$ to represent the impact of protein u (via reaction i) to up- or down-regulate protein d after treatment with compound k. Reaction i with protein u has the impact of up-regulating its target protein d in condition k ($u_{i,k}^{+}$=1) if $r_i . x_{u,k} = 1$; elsewise, $u_{i,k}^{+} = 0$. Similarity, reaction i with u has the impact of down-regulating d in condition k ($u_{i,k}^{-} = 1$) if $r_i . x_{u,k} = -1$. In any other case, $u_{i,k}^{-} = 0$. The variables $u_{i,k}^{+}$ and $u_{i,k}^{-}$ can be constrained as below:

$$u_{i,k}^{+} = \max(0, r_i . x_{u,k} - z_{i,k}) \tag{2}$$

$$u_{i,k}^{-} = \max(0, -r_i . x_{u,k} - z_{i,k}) \tag{3}$$

From formula (2-3), if reaction i didn't take place ($z_{i,k}$=1) after treatment with compound k, the protein u didn't have impact to change the protein d through this reaction ($u_{i,k}^{+} = u_{i,k}^{-} = 0$). The equations (2-3) can be represented with linear constraints (4-13) based on our previous work (DILP model) [2]:

$$u_{i,k}^{+} \geq 0 \tag{4}$$

$$u_{i,k}^{+} \geq r_i * x_{u,k} - z_{i,k} \tag{5}$$

$$u_{i,k}^{+} + 3d1_{i,k} \leq 3 \tag{6}$$

$$u_{i,k}^{+} + z_{i,k} - r_i * x_{u,k} + 3d2_{i,k} \leq 3 \tag{7}$$

$$d1_{i,k} + d2_{i,k} = 1 \tag{8}$$

$$u_{i,k}^{-} \geq 0 \tag{9}$$

$$u_{i,k}^{-} \geq -r_i * x_{u,k} - z_{i,k} \tag{10}$$

$$u_{i,k}^{-} + 3d3_{i,k} \leq 3 \tag{11}$$

$$u_{i,k}^{-} + z_{i,k} + r_i * x_{u,k} + 3d4_{i,k} \leq 3 \tag{12}$$

$$d3_{i,k} + d4_{i,k} = 1 \tag{13}$$

Hence, based on the sign of edge i and the state of start node $x_{u,k}$ under the condition k, $u_{i,k}^{+}$ and $u_{i,k}^{-}$ are calculated. When protein u is un-changed, $u_{i,k}^{+} = u_{i,k}^{-} = 0$; else wise, $u_{i,k}^{+} + u_{i,k}^{-} = 1$.

At last, the binary variables $x_{d,k}^{+}$ and $x_{d,k}^{-}$ were introduced to represent the impact (effect) for node d of being up- or down-regulated depending its parental nodes (upstream proteins) in condition k. Node d

has the impact of being up-regulated ($x^+_{d,k} = 1$) if $u^+_{i,k} = 1$ and node d has the impact of being down-regulated ($x^+_{d,k} = 1$) if $u^-_{i,k} = 1$. The state ($x_{d,k}$) of node d in condition k depends on $x^+_{d,k}$ and $x^-_{d,k}$ [2].

$$x^+_{d,k} \geq u^+_{i,k} \tag{14}$$

$$x^-_{d,k} \geq u^-_{i,k} \tag{15}$$

$$x^+_{d,k} \leq \sum_{\substack{w \in P, q \in E \\ u_w \xrightarrow{q} u_d}} u^+_{q,k} \tag{16}$$

$$x^-_{d,k} \leq \sum_{\substack{w \in P, q \in E \\ u_w \xrightarrow{q} u_d}} u^-_{q,k} \tag{17}$$

$$x_{d,k} \leq x^+_{d,k} \tag{18}$$

$$x_{d,k} \geq -x^-_{d,k} \tag{19}$$

$$x_{d,k} \leq 2x^+_{d,k} - x^-_{d,k} \tag{20}$$

$$x_{d,k} \geq -2x^-_{d,k} + x^+_{d,k} \tag{21}$$

According to formula (14-21), we can see node d may has the impact of being both up- and down-regulated if $x^+_{d,k} = x^-_{d,k} = 1$. The state of protein d cannot be accurately determined if $x^+_{d,k} = x^-_{d,k} = 1$.

The goal of next step is to remove the inconsistent reactions from the topology of a generic pathway map which do not occur in any treatment conditions, Therefore, the binary variable y_i denotes 1 if the reaction i is removed in the inferred cell-specific signaling network, and 0 else wise. The most important thing is that how to constrains the presence of reaction i in the inferred cell-specific pathway network through the state of reaction i in the condition k. We applied formulas (22-23) to address this problem [2].

$$z_{i,k} \geq y_i, \quad k \in C, i \in E \tag{22}$$

$$1 - y_i \leq \sum_{k \in T} (1 - z_{i,k}) \tag{23}$$

The state of $z_{i,k}$ may affect the fitting error between observed and predicted values of proteins. Formula (22) indicates that reaction i is present in the cell-specific pathway network if it takes place at least in

one condition. Formula (23) denotes that the reaction i is not included in the cell-specific pathway network if this reaction doesn't occur in all of the conditions.

To infer cell-specific pathway network, we applied our TILP approach with above constraints to minimize the differences between experimentally measured and predicted values of signaling proteins, as well as to obtain a minimized sub-network of original generic pathway map by optimizing the following objective function shown in formula (24):

$$\min_{X,Y,Z}\{\sum_{k\in T}\sum_{j\in P}(m_{j,k}-x_{j,k})^2+\gamma\sum_{i\in E}y_i\} \tag{24}$$

where three variable set *X*, *Y*, and *Z* denote all the variables $x_{j,k} \in X$, $y_i \in Y$, and $z_{i,k} \in Z$, respectively. Formula (24) indicates that more proteins are observed in experiment will increase the accuracy of optimization. Considering the fact that experimental data often cannot cover all of the proteins in signaling network, prior knowledge extracted from literatures was represented as linear inequality to constraint the expected value of $x_{j,k}$ and narrowing the search space. For example, we use $x_{j,k} \leq -1$ to express the fact that a target has been confirmed in the previous literature: the expression of protein *j* is inhibited after tumor cells were treated by compound *k*.

In the objective function shown in Eq. (24), the first term denotes the fitting error between observed and predicted values; and the second term indicates the number of interactions in the optimized signaling network. The observed and predicted values of *j*–th protein in condition k were denoted as $m_{j,k}, x_{j,k} \in \{-1,0,1\}$, respectively. When $m_{j,k}$ and $x_{j,k}$ are equal, the square error $(m_{j,k} - x_{j,k})^2$ is equal to 0; otherwise it is either 1 or 4. Hence, optimization of the above objective function might induce local-optimal solution because of the non-uniform distribution of the term $(m_{j,k} - x_{j,k})^2$. In order to address this bias, a binary variable $a_{j,k}$ (0 or 1) was designed as the difference between $m_{j,k}$and $x_{j,k}$ as following constraints (25-26): $a_{j,k}$ will be 1 if $m_{j,k}$ is not equal to $x_{j,k}$; the minimum of formula (27) will automatically set $a_{j,k}$as 0 if $m_{j,k}$ is equal to $x_{j,k}$ [2].

$$a_{j,k} \geq \frac{m_{j,k}-x_{j,k}}{2} \tag{25}$$

$$a_{j,k} \geq \frac{x_{j,k}-m_{j,k}}{2} \tag{26}$$

Then the square error $(m_{j,k} - x_{j,k})^2$ in above objective function was replaced by $a_{j,k}$.Therefore, the objective function (24) can be simplified to formula (27):

$$\min_{X,Y,Z}\{\sum_{k\in T}\sum_{j\in P}a_{j,k}+\gamma\sum_{i\in E}y_i\} \tag{27}$$

where three variable set X, Y, and Z denote all the variables $x_{j,k} \in X$, $y_i \in Y$, and $z_{i,k} \in Z$, respectively. By optimizing the above objective function, the predicted values for variable sets X, Y, and Z can be obtained. The negative constant γ in Eq. (27) is used to obtain a minimum sub-graph from the generic pathway map as the finalized cell-specific pathways (here, we have$-\frac{1}{|E|} < \gamma < 0$), in which $|E|$ is the number of reactions in the topology. In the optimization procedure, the predicted values of nodes and edges in signaling network meet our developed constraints.

In formula (27), minimizing the complexity of network structure by edge removals might eliminate some reactions, leading to some phosphpor-signals may not be transduced from upstream into downstream (see one example shown in **Fig.6**). Given a generic pathway map as shown in **Fig.6A**, we can find that **Fig.6B** and **6C** will result in equal fitting precision between predicted and observed values. However, "missing edges" in **Fig.6B** leads to interruption of the signal from upstream. Therefore, the solution shown in **Fig. 6C** is optimal in this case. To address the problem mentioned above, we further defined two constraints (see formula (28-29)) to avoid missing edges in the optimization process.

$$-1 \leq x_{u,k} \cdot x_{d,k} + z_{i,k} \leq 1 \tag{28}$$

$$-1 \leq x_{u,k} \cdot x_{d,k} - z_{i,k} \leq 1 \tag{29}$$

Formula (28) indicates that the activation reaction i under condition k takes place if $x_{u,k} \cdot x_{d,k} = 1$. Similarity, the inhibitory reaction i under condition k take place if $x_{u,k} \cdot x_{d,k} = -1$ (formula (29)). In addition, if there are several upstream proteins connected to the same downstream protein, the constraint shown in formula (30) ensuring at least one reaction occurred.

$$\sum_{h=1}^{H} z_{h,k} \leq H - 1 \tag{30}$$

where $z_{h,k}$ is the h-th reaction under condition k, and H is the total number of reactions connected to a downstream protein.

The formulations in TILP presented above were implemented in a Matlab-based optimizer, GUROBI 6.5.1, which is a well-known mathematical programming solver. The optimization of formula (27) will deliver an optimal sub-network of the generic pathways which can best explain the experimental data.

S2. Computational procedure: fitting precision of data (goodness of fit)

In the process of searching the optimal cell-specific pathways, we defined fitting precision (FP) to describe the goodness of fit between the predicted values derived by our TILP approach and the measured values of proteins under all the time points.

$$FP = \frac{\sum_{j=1}^{N}\sum_{k=1}^{T} a_{j,k}}{N \times T} \times 100\% \quad (31)$$

Where the binary variable $a_{j,k}$ indicates the difference between the measurement $m_{j,k}$ and predicted value $x_{j,k}$ of j –th protein in condtion k. N and T are the total number of measured proteins and treatment conditions, respectively. The value of fitting precision (FP) is in the range from 0% to 100%.

References

1. Ji Z, Su J, Liu C, Wang H, Huang D, Zhou X: **Integrating genomics and proteomics data to predict drug effects using binary linear programming**. *Plos One* 2014, **9**(7):e102798.
2. Ji Z, Wu D, Zhao W, Peng H, Zhao S, Huang D, Zhou X: **Systemic modeling myeloma-osteoclast interactions under normoxic/hypoxic condition using a novel computational approach**. *Sci Rep* 2015, **5**:13291.
