# Supplementary material for: A linear programming computational framework integrates phosphor-proteomics and prior knowledge to predict drug efficacy
Source: BMC Syst Biol. 2017 Dec 21;11(Suppl 7):127. doi: 10.1186/s12918-017-0501-6 (PMC5763468; doi:10.1186/s12918-017-0501-6)
Supplement: Supplementary file 6 — The response network induced by verapamil. (PDF 476 kb) [file 12918_2017_501_MOESM6_ESM.pdf]

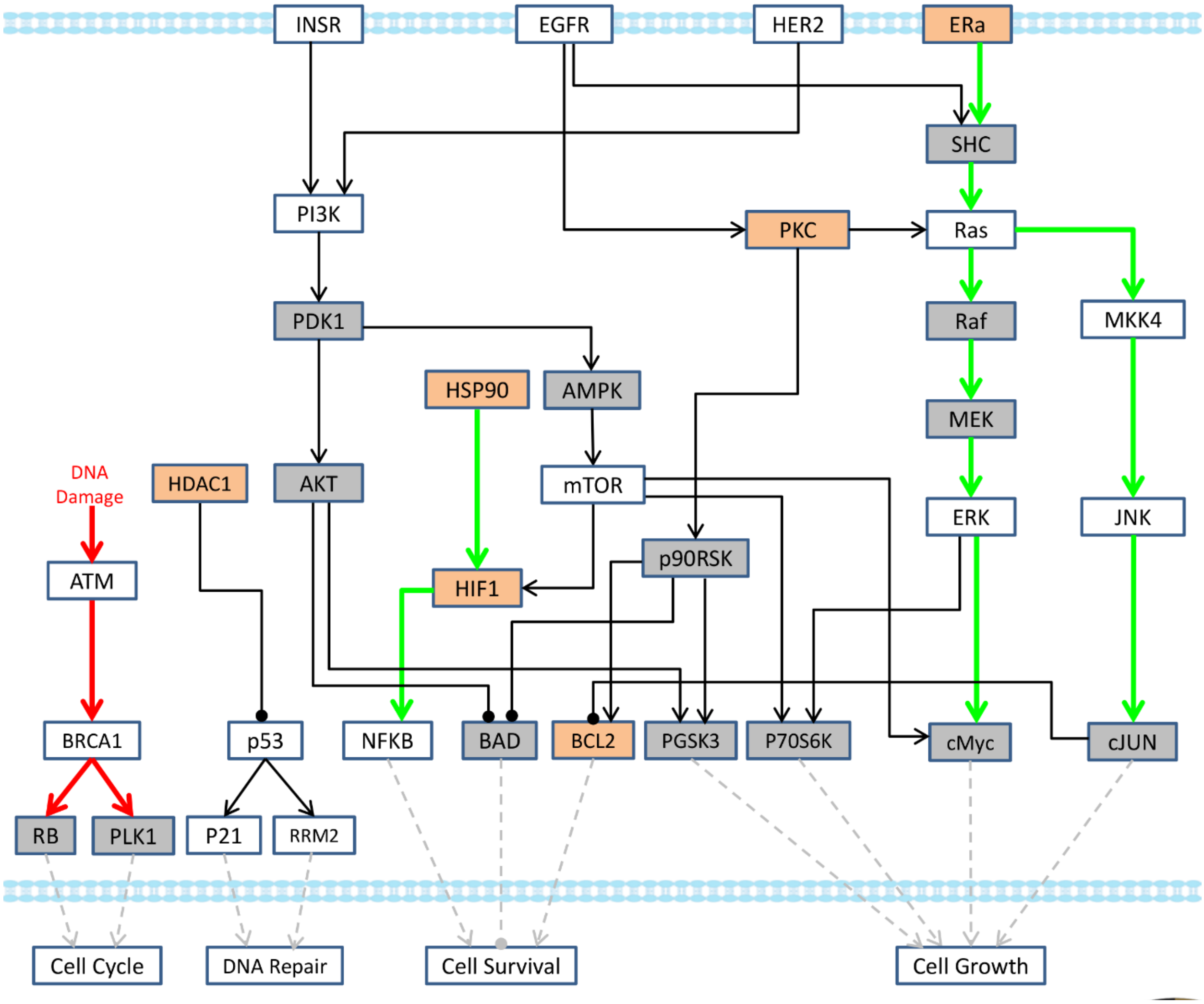


**Fig. S5** The response network induced by verapamil. A pathway with red color was up-regulated, green color was down-regulated, black color was un-changed. The edges with yellow color were removed after optimization.
